# A Report on Typhoid Fever, Made to the Scott County Medical Society

**Published:** 1850-11

**Authors:** W. L. Sutton

**Affiliations:** Georgetown, Ky.


### WESTERN JOURNAL

OF

### MEDICINE AND SURGERY.

NOVEMBER, 1850.

ART. I.—*A Report on Typhoid Fever made to the Scott County Medical Society*. By W. L. SUTTON, M. D., of Georgetown, Ky.

(CONCLUDED.)

### CHAPTER VII.

### ARTICLE 1.

### TREATMENT—BLOOD LETTING.

There appears to be a notion amongst many physicians that when we have determined that a given case is one of typhoid fever, it is to be let alone and no attempts made to control it—as in this age of progressive improvement, when we have named our children we suffer them to pursue their own course without restraint. One conclusion is about as sage as the other. By ill-tempered, rash, and inconsiderate conduct we may do immense injury in either case. On the contrary by great care, vigilance, prudence, and firmness, we may do much good.

I wish it was in my power to give plans of practice, better settled than any I have seen yet, as also something which would promise more success than usually attends our best endeavors. Nor have I the consolation to know, that among those who have been long familiar with the disease, there is a greater unanimity as to the best mode of treating the complaint, or a more fortunate result in their treatment, than may be found in this vicinity. I am ready however to give what seems to be the treatment in this section, according to my own personal experience, aided by the observations of my professional brethren, with whom I have made it a point to converse freely whenever opportunity offered. In delineating the course of treatment I shall take up one means after another, and first as to

*Blood-letting.*—My experience and observation as to this remedy, are both very limited. I have myself bled but one patient in this disease, and that was under a false diagnosis. The pulse indicated that it would *bear* the lancet though it did not *demand* it. The consequences were very unpleasant; continued prostration with repeated syncope gave me much uneasiness. The case however terminated favorably. In another patient of mine, in which there was considerable headache, and some firmness of pulse, bleeding was performed in my absence, without any marked effect save a temporary abatement of the headache. The patient did not seem to be injured by the operation, but the case eventuated unfavorably. In a third, late in the disease when the pulse was over one hundred and thirty in the minute, profuse epistaxis having come on, bleeding to something more than a pint was instituted for the relief of that symptom. No benefit resulted; I saw the patient next day, the hemorrhage was stopped, not however by the bleeding, but death followed at the period of four days from the bleeding. In a fourth during the third week a patient was bled to a small amount say f℥iii upon some increased fullness of pulse, occasioned probably by the action of quinine. I saw this patient for the first time the day before he was bled, and did not consider him beyond remedy. The day after the bleeding, I saw him again, when it seemed to me he was so. It is certainly true however that he was in a condition at my first visit which did not promise much.

I do not conceive that I have anything to do with the practice in other countries, else it would not be difficult to find apparently strong evidence, that blood-letting is an agent of great value. I would also refer to the amount of blood sometimes lost from the nose or bowels in the latter stages of bad cases of fever, where yet the patient recovers. Although I am not prepared to say in what the effect of hemorrhage differs from that which follows blood-letting to the same amount, or whether it does differ, I have thought it my duty to allude to it. I will also take occasion to say here, that this consideration has been weighed with much interest, viz.: *Inasmuch as considerable hemorrhage sometimes takes place in advanced stages of very bad cases, and the patients yet recover; is not this an evidence that venesection was called for in the early stage, but that call not appreciated?* Such interpretation will be given readily by one who thinks blood letting an important agent in the disease. Such may be the proper view. But I would observe that, so far as my observations go, hemorrhage is much more apt to occur in some families than in others. For instance, of forty-three patients seen in the last twelve months, considerable hemorrhage occurred in six cases. Of these, two occurred in one family, they being the only cases in that family; and four in another family in which there were nine cases.

In this second family, there was a patient who had a pulse decidedly fuller than is usually found in this fever, at least in this vicinity; one which would have justified bleeding. It however was not resorted to. During the continuance of this case, I reflected much upon this point. If hemorrhage occur in this case, how much wei_ht is to be given to that circumstance, as indicating the necessity of blood-letting at the commencement? It appeared to me then, and it does yet, that the formation of a proper conclusion would have been attended with much difficulty. That however was one of the cases in which no hemorrhage occurred.

Again one of the patients at Mrs. Lightburn's presented a pulse of much the same character; and I caused him to sit up, intending to bleed him if his pulse retained its fullness in the sitting posture; his pulse however flagged upon rising, and he was not bled. Again, in one of the cases at Mr. Cooper's in 1841, the pulse was fifty per minute and full, such as would seem, under ordinary circumstances to *demand* bleeding. It however was not performed. Each of these cases had a speedy convalescence.

Topical bleeding is less liable to objection. In many cases where there is much pain in the head or abdomen, abstracting blood by cups may be practised with much advantage.

---

### ARTICLE 2.

### EMETICS

May be used with a very reasonable expectation of benefit in the early stage of the disease, when there is nausea or oppression of stomach, when there is a yellowish tinge upon the tongue, or a bitter taste in the mouth. During the progress of the disease an emetic may be administered when there appears to be a considerable redundance of bile or phlegm, by which the stomach is oppressed. It is however always to be borne in mind that when diarrhea is present to a marked degree, we cannot depend upon an emetic operating well, how desirable soever it may be. In such cases, it is always apt to run off upon the bowels. Even in the advanced stage I have seen an emetic which acted mildly do much good, but it has been when there was not much diarrhea.

On the other hand, an emetic of tartrite of antimony and ipecac, administered on the tenth day of disease, where there had been much watery purging, failed to vomit effectually, but ran off by the bowels, producing great prostration, serous bloody stools, &c. Death followed as a consequence.

When there is much diarrhea therefore, we should not venture upon the use of emetics without very cogent considerations, and under all circumstances we should watch their effects with great care. Of course it would appear advisable to select an emetic which is not apt to affect the bowels—therefore we prefer ipecac to tartar. I do this even when no diarrhea has appeared for fear it should be excited by the operation.

---

ARTICLE 3.

CATHARTICS

Are very important agents in the treatment. If an emetic has been administered, it should be followed at the interval of twelve or twenty-four hours by a cathartic. Unless there should be something to forbid the use of mercury, I almost always give calomel or blue-mass in the first purgative. If there is any disposition to diarrhea, I generally combine with ten grains of calomel as much Dover's powder; or six grains of calomel, as much blue-mass, with the Dover's powder, or its equivalent of morphia and ipecac. Some physicians think that in cases where diarrhea is present blue-mass is peculiarly appropriate; that it checks the diarrhea. That this last is many times true there is no doubt. But the same is true of calomel. On the contrary, in many instances there is no marked diarrhea until an emetic or cathartic is given; hence some object to the use of medicine because it is apt to produce this state of things. But the truth is, that if diarrhea has not appeared before, it is very apt to occur about this time; whether medicine is administered or not. We therefore give a cathartic combined as above without much attention to the presence or absence of diarrhea. If there is diarrhea when the first medicine is administered, and it should not be checked by it, it should be repeated at an interval of twenty-four or forty-eight hours. If it still continues, small doses of blue-mass with morphia and ipecac. say pil. hydrarg. gr. iii, ipecac and opii aa. gr. $\frac{1}{4}$ or an equivalent of morphia in lieu of the opium, to be repeated every four or six hours as necessity may indicate, will be proper. If there should appear a deficiency of bile, or a derangement of its quality, this will be the more proper. In such cases too, an occasional dose of grs. x or xii of blue-mass will be called for during the course of treatment, and that though there may be continual necessity to restrain the bowels. If under this treatment, the dejections are more consistent with an admixture of bile, we will consider ourselves as making a favorable impression upon the disease. At any rate, it is desirable that the bowels be moved decidedly though gently, in the first two or three days of the disease.

If there should have been no diarrhea, or indeed if there should be, and the bowels should not be moved in eighteen hours after the administration of the purgative, it may be well to give a small dose of rhubarb, castor-oil, or epsom salt. Or if there should be reason to think that there are worms in the bowels, some oil of wormseed may be added to the castor-oil, provided we have a good article; if not, Fahnestock's vermifuge may be substituted for the oil, or an infusion of senna and spigelia may be used.

It is to be remembered however, that any of these adjuncts may act more powerfully than is desirable. Indeed three Seidlitz powders taken in the course of the day succeeding that on which a mercurial had been administered, produced a couple of satisfactory stools, and afterwards caused much griping with watery bloody stools, which it took two or three days to rectify. The same state has followed the administration of rhubarb given as an assistant to calomel. We should always therefore, watch our remedies carefully and be prepared to interfere, when they begin to act unkindly.

Here I would make a few remarks upon the use of calomel in this form of fever, as compared with its use in bilious fever. In this latter disease, if our calomel was followed by serous purging, we increased the dose with confidence to ℈i ʒss, ʒii, or in some few cases of unusual obstinacy to ʒi. This is the largest dose I ever gave, and that perhaps not a dozen times in a period of thirty years practice. We confidently expected to effect by this course, consistent, bottle-green, bilious stools, and as confidently an amendment of the patient under these discharges. Indeed, in a bad case it was not usual to see amendment until an impression resulting in this kind of purging was made. Under this conviction I have at divers times, when I found my patient evidently better, and no purging had taken place, predicted with confidence, that such purging would ensue in the course of the next twelve or twenty-four hours. Nor do I remember a case in which the prediction was falsified.

Here however, the state of things is different. We cannot give large doses of calomel with the assurance that consistent stools will follow—at least I never tried it except in two cases. In those ℨi of calomel only increased the watery purging which followed smaller doses. Neither have I in any case of typhoid fever, seen calomel in any dose, produce the consistent bottle-green, bilious stools which we consider so favorable in bilious fever. I have seen dark green stools, but they have been mixed to a considerable extent with mucous. The green too is of a shade materially different, they also have a shining glossy appearance, altogether different from those before mentioned. Neither does amendment have any connection with the appearance of stools of this kind, on the contrary patients may, and frequently do get worse during their continuance.

In a certain proportion of cases, a suspended secretion of the liver attends this fever, which accompanied by a state of irritation in the mucous membrane of the bowels, causes a discharge of considerable quantities of whitish mucous. Here small doses of calomel or blue-mass with opium and ipecac are again called for.

Although a vitiated or suspended secretion of bile is present in a considerable proportion of cases, it is by no means necessary to the existence of the disease. On the contrary, in many cases though there may be considerable diarrhea yet the admixture of bile appears to be in fair quantity. The fault being in the excess of watery particles, not in a deficiency of bile. The excretion is excessively fetid to be sure, but that fetor is acquired below the duodenum. Further, many cases run their course, with very little variation in the character of the stools for a long time; at length mixed in this watery discharge, a very few minute portions of consistent, apparently well elaborated feces make their appearance. These gradually increase in size and number, until as convalescence advances, the feces assume more and more a healthy consistence and aspect. In cases of this kind, in which there would seem to be no marked deviation in the quantity or quality of the bile, mercury, as a chologogue at least, does not seem to be demanded. It is not necessary to stimulate the liver when that organ is already doing its duty.

Calomel in doses of fifteen to twenty grains has been given for the purpose of arresting hemorrhage from the bowels, and it is said successfully; blue-mass, opium, and ipecac, as recommended for mucous stools have likewise been given for the same symptom, with satisfactory results. And in some cases although the hemorrhage was considerable, it has been let alone, and that too with success. The truth is, I do not think we understand the cause and import of this symptom. If it occurs, as there is good reason to think it sometimes does, from the erosion of a considerable vessel, we shall probably be unable to control it by any means; if it is from the capillary vessels of the mucous membrane, it may relieve the congested vessels or be in some degree amenable to medicines. When in moderate quantity, and indeed in some cases when it could not be considered moderate, it has not appeared to injure the patient at all. Yet it is an ugly symptom and one which may prostrate the patient very speedily.

When it becomes necessary to open the bowels in the latter stages of the disease, injections of warm water should be used for that purpose, unless blue-mass or some other laxative should seem to be called for. In that case we should still be on our guard, lest whatever is given should irritate the bowels improperly.

### ARTICLE 4.

### MERCURY.

Of *mercury*, given to produce its constitutional effects, I know little. I have never given it for that purpose. In some cases I have had a sore mouth to appear during treatment. In fact I have thought that effect rather easily produced during this fever. I have seen this state continue a week, the patient all the time growing worse. Again, I have seen the patient improve during its continuance, without however being at all satisfied that convalescence was affected in any degree by its presence.

---

### ARTICLE 5.

### TONICS AND STIMULI.

Where there is a perceptible periodicity, quinine has appeared to be decidedly beneficial in arresting the paroxysms and mitigating the severity of the disease; but I do not know that it can be depended on to shorten its duration.* It is true however that it has sometimes *appear-*

---

*I am inclined to think that there is frequently a periodical depression in this disease which escapes observation, *e. g.:* Whilst sponging a patient who complained of warmth, and whose forehead felt warm to my hand, I observed a very perceptible lividity of the lips and also of the nose; this lividity indeed was perceptible in the countenance, but would perhaps have escaped observation had it not been for the nose and lips. The toes were very slightly cool. This was all the evidence of depression. Acting upon this hint, I gave ten grains quinine a few hours afterwards and repeated twice a day for several days, with, apparently, the most unequivocal good effects.

*ed* to have that effect; and some of my medical friends have a considerable confidence in its powers in this particular. In the latter stages, when the powers of life begin to flag, it may likewise be administered with benefit. It has appeared to me that under these circumstances, a combination with a small portion of blue-mass adds very much to the beneficial effects of the quinine. Dr. Desha places much reliance upon quinine combined with calomel or blue-mass, as an antidote for profuse diarrhea. I have used calomel gr. xii with quinine gr. v, or a similar dose in several instances with much apparent advantage. I have used it too where no benefit resulted from it. Altogether I have a favorable opinion of the prescription and think it well worthy a fair trial. It may not be altogether irrelavent to remark that in 1833 during the cholera, I thought nothing insured a satisfactory action from calomel so certainly as a combination with it, of five or ten grains of quinine. For this condition of the bowels Dr. Barlow has used with much satisfaction the tinct. camph. in doses of a teaspoonful.

I have seen, since writing this essay, some cases in which quinine appeared to have a most happy effect, *e. g.:* Mrs. G., February, 1849, had taken a small dose of calomel and some smaller doses of blue-mass, ipecac, and opii, without any apparent benefit. Having a profuse watery diarrhea, frequent small pulse, tongue thickly coated and dry, extreme muscular weakness, incoherence; she took quinine grs. v in the morning and again in the evening. After that grs. x every morning for several days. The dose was then gradually diminished. No other medicine. Under this course the diarrhea diminished and eventually constipation was established so that she had no stool for six days, when her bowels were moved by injection; the tongue improved gradually, the pulse came down, and her strengih increased, and she had a regular though tedious convalescence.

Stimuli in the form of wine, brandy, or whisky, have seemed to exert a very beneficial influence in a few cases, those cases however have been but few. Whether their use was delayed too long, or I was unfortunate in my selection of cases, I know not, but upon the whole I have no great cause for gratulation. Some of my brethren however speak much more encouragingly of their use. In one case I used whisky pretty freely, where there was a distinct pulsation of the heart, one hundred and forty to the minute, with a weak one at the wrist seventy to the minute. It did no good. This was the case before mentioned, in which the emetic operated so unfortunately. Was it a case adapted to stimuli? Generally I have considered a weak impulse of the heart as indicating a necessity for supporting the powers of life.

---

### ARTICLE 6.

### APPLIANCES TO THE SKIN.

SECTION 1. *Blisters.*—When there is considerable pain in the head or abdomen, when venesection or cupping has been used if thought advisable, or the time at which either would have been allowable has been lost, a blister to the nucha or abdomen as the case may be, can be used with much promise of advantage. Upon my first acquaintance with the disease, I used blisters on the abdomen in nearly every case where there was any decided tympanitis. Such I think is the practice with many physicians. I have for several years, ceased their use for that symptom, for two reasons: One is, I am not sure that I have in any instance seen a decided advantage from their employment. Secondly, on account of their inconvenience. They occasion a considerable annoyance in the various motions of the body, and in the frequent risings to stool, the dressings are very apt to become deranged. But the chief inconvenience arises from their liability to produce stranguary. And I think this liability is decidedly greater in typhoid fever than in most diseases. As intense a degree of suffering as I have ever witnessed was from the re-application of a blister. I therefore disregard tympanites as an index, and am guided by pain; and sometimes when there is a torpor of the liver I apply one over that, as for the same symptom in other diseases. Occasionally we may take advantage of a blistered surface to apply morphia to it when there seems to be occasion for it, and we do not like to give it by the mouth or anus. I have seen a grain of morphia sprinkled upon a plaster of cerate and applied to a blistered surface induce sleep when there had been great want of it. I have likewise seen restlessness sensibly abate under its use. We should remember, however, that morphine, used in this way, will sometimes affect the system as sensibly as if given by the mouth.

Sec. 2. When we should fear ill effects from the application of a blister, or the occasion may seem to demand some counter irritation, but yet the blister may seem unnecessarily severe, or there may be sufficient reason to think that it may not be necessary to keep up the irritation for a considerable period; sinapisms offer themselves as a suitable substitute for the blister. They may be used repeatedly on the same or adjacent spots, as long as may be deemed advisable. They have one advantage in requiring no subsequent dressings. It has appeared to me however, when I have applied a blister on a surface which had been acted on by sinapisms, that the blister did not draw as well as upon a surface to which no sinapism had been applied—unless indeed, it was applied pretty soon after the removal of the sinapism. My explanation of this circumstance is, that the sinapism produces a state of the skin similar to that produced by a slight burn. A milder remedy for the same purpose, yet one by no means to be despised in abdominal uneasiness, is a poultice applied as circumstances may require.

Sec. 2. Inferior to no remedy in this disease is the external application of water, cold or warm, as the case may require. When there is severe pain in the head, a stream of warm water poured for some time on the forehead will be found very efficacious in relieving it. Cold water applied in the same way or by cloths, will in many cases answer equally well. When there is much pain or heat, or both in the bowels, cloths wet with cold or warm water, especially the former, and kept constantly applied, will be found both grateful to the feelings and serviceable in relieving the symptoms. Free and frequent sponging with cold water will be found particularly efficacious in reducing the heat of surface and febrile activity of the pulse.

Sec. 3. *Pediluvia.*—As there is great tendency to coolness of feet and knees, bathing the feet and legs in warm water two or three times a day, is often followed by much advantage. When there is much muscular prostration, and the patient is unable to bear the exertion of sitting up, he should be turned across the bed with his legs over the side, and his feet in a bucket of warm water, elevated to a proper height. When replaced in bed, the feet should be wrapped in warm blankets or have bottles filled with warm water or a warm brick applied near them. These warm bodies ought to be renewed whenever the extremities become cool. When there is much pain in the back, loins, or even in the limbs, it may very frequently be removed by rubbing the part with laudanum, or equal parts of laudanum and ammoniated alcohol.

### ARTICLE 7.

### DIAPHORETICS.

As there is generally a marked dryness of the skin, it would seem probable that this class of remedies would be particularly called for. Our anticipations have not been realised in their employment. And when a decided moisture is observed upon the skin, improvement does not always follow. Yet it certainly is desirable to keep the skin soft and as near the natural condition as possible. For this purpose Dover's powder or a mixture of morphia and ipecac. is perhaps oftener used than any other formula. Would the old prescription of spt. mindereri with vin. antim. and spts. nitre be beneficial? It is adapted to many forms of fever, particularly when there is some tendency to irritability. No diaphoretic is better than frequent ablutions with cold water.

---

### ATRICLE 8.

### OPIATES

I have generally found valuable in the treatment of this fever. As mentioned when speaking of purgatives, I have generally found it proper to combine them in some form with the purgatives used at the beginning of the disease, to obviate irritability of the bowels. They are also sometimes necessary to procure sleep or remove restlessness. When it is necessary to give them by the mouth for this purpose, especially towards the commencement of the complaint, it is proper to combine them with some febrifuge to obviate their stimulating effects. When it is necessary to restrain diarrhea, that is when there are more than four watery discharges of moderate size in twenty-four hours, or larger, but fewer stools, amounting in all to more than a quart, we can give by injection from fifteen to thirty drops of laudanum in two ounces of warm water or thin starch, after each stool until they are reduced within due bounds. It sometimes happens that the bowels are too irritable to admit the retention of even that small bulk; in that case, half a grain of opium added to enough castile soap to make a common sized pill, introduced up the rectum, will be found a valuable expedient. For stranguary, whether arising spontaneously, or brought on by the application of a blister, I know of no remedy better than opium. This symptom may occur when there is occasion to restrain the bowels, or when the bowels do not require interference, or when it may be necessary to move them. In the first or second instance, from twenty to sixty drops of laudanum, according to the urgency of the case, in two or three ounces of warm water thrown into the rectum will almost always relieve it. If the bowels require to be moved, then the same quantity in half a pint of warm water will be advisable. Should laudanum administered in this way fails we must give it by the mouth in similar doses.

---

### ARTICLE 9.

### EXPECTORANTS

Are sometimes, though according to my observation rarely, necessary. Then a weak watery infusion of ipecac with enough paregoric or morphia to prevent its running off by the bowels—a mucilaginous mixture, with small doses of wine of ipecac or antimony—infusion of senega with liquorice, with or without a minute opiate, will be appropriate.

ARTICLE 10.

DIET AND DRINKS.

SECTION 1. *Diet.*—The appetite being usually destroyed, there is little need for nourishment, indeed it will frequently be difficult to induce the patient to take as much as is proper. Nothing but the most bland articles should be allowed, and that in small quantity. Three or four teacupsful of arrow root in the course of twenty-four hours are sufficient, or if the bowels are disposed to costiveness, the same quantity of gruel well made. It will frequently be necessary to change these articles, one for the other according to the state of the bowels. As the disease advances, and the strength of pulse begins to give way, this diet may be seasoned with brandy or wine and nutmeg. This bland diet should be continued until convalescence is fairly established. There is no need to fear that under this course "the patient will never get his strength," as is sometimes said by the patient or his friends. After amendment has continued some days, the appetite frequently becomes pretty good, sometimes ravenous. Great care now becomes necessary to prevent injury from excess or improper food. There will be great disposition to indulge in improper articles. Some increase in the quantity of the bland food before mentioned, may now be allowed for two or three days; then something a little more substantial, as mush and milk, the milk being boiled or not, according to the state of the bowels—gradually, very gradually, returning to the ordinary diet. We should remember that we have had to do with a very grave affection, one distinguishing feature of which has been derangement of the digestive apparatus. We should infer that this apparatus will remain in a weak, irritable state for a considerable time, and therefore nothing should be presented to it, which by either quality or quantity would be in any danger of reproducing that irritation.

Sec. 2. *The drink* should be mucilaginous; but as most persons will become tired of one kind, when continued long, we find it necessary to change it occasionally. I do not know that there is a material difference between cold and warm drinks. Many physicians allow their patients to choose for themselves. For myself, when there is not a decided tendency to diarrhea, I have no choice; I permit any drink in moderation, which the patient may desire. Where there is a decided disposition to looseness, I prefer the drinks to be warm; or if there is no satisfying the patient with warm drinks, I allow water to be boiled and then cooled to the temperature of spring water. I am not sure that this is not a matter of fancy: my reason, however is this—I have thought that our limestone water was rather apt to aggravate irritable bowels. I am of opinion too, that I have seen cold water, ice water, bring on diarrhea: wherefore I prefer that the water be boiled when this state of the bowels is present. Sometimes I choose to administer carbonate of soda or carbonate of ammonia in the drink as a means of removing the irritable condition of the bowels, or of supplying a mild stimulus; when we choose to administer the ammonia, we should remember that if it is added to the water above blood heat, the salt is decomposed.

When the disease is marked by decided mildness of symptoms, it is well to abstain from medicines and depend upon diet and regimen exclusively. It is very important, however, and sometimes very difficult, to distinguish between these mild cases, and those in which the disease increases very gradually, but steadily. These last are frequently the very worst cases we have to contend with.

### ARTICLE 11.

### MANAGEMENT OF PATIENT.

So far is it from being true, that this disease is a sinecure to a physician—that he has nothing to do—that I know of no disease fraught with more solicitude and anxiety. It is true we do not think powerful doses of medicines called for, nor very active treatment proper; but the duration of the disease and the various points which, however important, appear to many to be small matters, yet

'Make up in number what they want in weight.'

We should therefore reflect that we have to manage a case of considerable duration; and that how mild soever it may be at the onset, we have no assurance that it will continue so; and we must make our arrangements accordingly. We should select a room for our patient which is large and airy ; one from which air and light may be excluded at pleasure, and little exposed to noise. Little furniture may be required: a bed for the patient, and something upon which the nurse may occasionally recline, a couple of chairs, one for the nurse and one for the physician during his visits, a table upon which such articles as are needed, as medicine and drinks, may be placed, suitable implements for warming drinks, &c., are all that are necessary. In the winter, a carpet at least by the bedside, will be proper. Indeed I do not consider a carpet will be objectionable at any time. It will tend to prevent noise in the room. The bedstead should be of a medium height, between the ordinary and the truckle bedstead, as more convenient to a patient of considerably diminished muscular power.

One of the most important things to the patient, and second only to the physician, is a suitable nurse. He should be kind, little disposed to talk, cheerful, decided in his temper, one who will have the control of the patient, and who will show to all who are disposed to meddle, that he *will have* the management of the case. As it will probably be necessary to administer injections repeatedly during the disease, he should understand the composition of them as given for the different intentions, and be qualified to administer them without pain or danger of irritation. He should have a light hand so as to enable him to dress blisters or do other necessary things, with the least annoyance to the patient. He should speak cheerfully and encouragingly to the patient; and although he should be civil to visiters, it would be well if he could give needless ones to understand that too much company is not desirable. Some persons visit the sick that they may soothe their uneasiness, or show a disposition to do kind offices for them either in the sick room or without; many more visit the sick, that they may meet some one there to talk to, or to lounge away their time. The first require no accommodation, the second deserve none, and the sooner they are dismissed the better.

The temperament and especially the wishes of the patient respecting the admission of company or not, should be consulted. Some persons when sick, dislike to be annoyed by the presence of company, to such the less company is admitted the better. Others again have their spirits considerably enlivened by the presence of their friends; in such cases occasional visits by persons for whom the patient has a fondness need not be forbidden. But more than one or two should never be admitted at the same time. All gloomy conversation ought to be strictly interdicted; also all allusion to persons who have lately died, or such as are dangerously sick. Although the conversation should be cheerful, it should not be frivolous or light.

Each evacuation, whether by vomiting or purging, should be removed immediately. The bed and body linen should be changed frequently; the face and hands washed; and the head combed daily; the mouth rinsed every few hours when there is an unpleasant taste in the mouth; and when there is much dryness of mouth, it should be moistened as often as is necessary with some warm slightly demulcent fluid. All noise should be excluded; and all light at all oppressive to the eyes; but air in pleasant weather should be admitted freely, yet in such way that no current shall fall upon the patient. The offensive odor from the excretions or from the body, may be masked by burning paper or sugar in the room when the weather forbids a sufficient admission of air to carry it off. In addition to sponging the body with water, in hot weather, the patient may be rendered more comfortable by wetting the floor repeatedly in the course of the day.

When the disease is very grave, every exertion possible should be avoided. Many times, when it would seem from the feelings of the patient, and indeed from his symptoms, that he might be able to walk about the room, rising to stool will produce a prostration from which some time will be required for recovery. In such cases he should not be permitted to rise. It will not do to encourage him to rise under the belief that the debility is fancied, rather than real.

All topics at all calculated to excite his mind must be excluded, and the mind kept perfectly quiet. Even when improvement has progressed considerably, exertion of both mind and body should be prevented as much as may be—divers persons have been supposed to be considerably advanced in amendment, who, upon transacting some business matters, have suddenly become worse, or even expired almost immediately.

### CHAPTER VIII.

### ARTICLE 1.

### CASES.

To make my delineation of this disease as intelligible as possible I think proper to add a few cases of the disease—first, of some which terminated favorably, then of some that ended fatally.

*Case* 1st.—Ellen, colored, aged thirteen years, has been complaining for several days; took her bed 3d instant. Saw her on the 5th. Complains of universal muscular soreness and debility; considerable abdominal tenderness; headache; sore throat; tongue covered with white fur with red points appearing through it; pulse, lying, 100, sitting 120; tremulous motion of the lips; some diarrhea. ℞.—Calomel grs. x, pulv.; Dover, grs. v, *statim.*

6th. Four or five stools; less muscular soreness, but abdominal tenderness unabated; meteorism; skin soft, rather warm; pulse 90; throat still sore; tongue as yesterday. ℞.—Emp. episp. abdomini. Pil. Hydrarg. grs. xi, opii gr. i, hor. decub. Pediluvia.

7th.—Much as yesterday as to pulse, skin, abdomen, and tongue; has had several watery putrid stools. Blister did not draw well. ℞.—Reapply blister; repeat the pill at bed-time; gargle of capsicum.

8th.—Somewhat delirious last night; six stools of same character, one contained a lumbricus; feels better; throat not so sore; tongue disposed to cast off fur, but is left very red; meteorism and tenderness unabated; skin and pulse as yesterday. Blister drew well but does not discharge well. ℞.—Injection of starch containing fifteen drops of laudanum after each stool.

9th.—Delirious last night, and occasional pain in the belly; six stools, somewhat more feculent, same fetor; two lumbrici; meteorism and tenderness of abdomen unabated; pulse, skin and tongue as before. ℞.—Remove the cuticle from the blister. Pil. Hydrarg. grs. v, pulv. Dover grs. ijss. Continue the injections. Arrow-root exclusively for diet; mucilage for drink.

10th.—Stools said to be less offensive, otherwise same as yesterday; meteorism and tenderness much less; tongue less red; skin warm and pleasant; pulse 82; less delirious last night; discharged a lumbricus from the throat; blister drying; took a pill of blue-mass and Dover's powder by mistake last night; was thought to be easier after it. ℞.—Reapply the blister; continue the arrow-root; and continue the injections provided the stools are more frequent than once in six hours.

12th.—Three stools in twenty-four hours; tenderness in iliac region; pulse 81; tongue reddish, smooth, and moist; some delirium last night. ℞.—Pil. Hydrarg. grs. v, to-night.

14th.—Pulse, asleep, 74, awake 78; tongue much as yesterday; skin soft; less delirium last night; one stool, less watery. Forgot to take the pill last night. ℞.—Pil. Hydrarg. statim; the blister to be kept discharging.

15th.—Pulse 78; tongue and skin as yesterday; three stools; still considerable tenderness in right iliac region; none elsewhere; no delirium; gums slightly sore; craves food. Continue the arrow-root and pediluvia. Continued to improve—discharged on 20th.

It is to be observed that the pulse on the 5th was 100; and on the 6th 90. This is to be explained, as I presume, by the fact that my first visit was made in the afternoon, and my subsequent ones in the forenoon. It is altogether probable that for several days the pulse in the afternoon was as frequent as on the 5th, as there is usually an increase (especially in the early stage of the disease) of from five to fifteen strokes in the afteruoon.

*Case* 2d.—Mrs. R. aged forty-one, was badly salivated ten years ago. Since that time has been in rather delicate health. Was considerably indisposed on the 27th of November, 1845, which increasing, I saw her on the 30th. Great suffering from pain in the head and back; right cheek flushed; respiration somewhat quickened and irregular; expiration rather sudden; no pain of chest; pulse 100; tongue slightly red and moist; skin rather dry; tinnitus aurium; slight nausea; some tenderness on pressing the epigastrium and right iliac region; abdomen scarcely distended. Took this morning a teaspoonful of table salt, which has moved the bowels three times, the stools liquid and of a putrid odor. ℞.—Calomel, Dover's powder a. grs. x, to be followed by small doses of sulph. magnesia at intervals during the night, provided there is any considerable fever; if not, and the calomel does not purge by 9 o'clock to-morrow, sulph. magn. ℥ii; laud. and alcohol, ammon : in equal quantities, to be rubbed upon the back; warm bathing to forehead.

Dec. 1, 1 P. M.—Pulse 82; tongue slightly red; skin as yesterday; pain in back and head much diminished; slight pain in neck and breast. Medicine has operated but once, thin and fetid. ℞.—Infus. senna to assist the medicine.

2d. 1 P. M.—Medicine operated five times, thin, fetid; tinnitus aurium considerable; little pain in either back or head; pulse 72; tongue as yesterday; soreness of abdomen. ℞.—Mucilage with super. carb. soda.

3d. 3 P. M.—Pulse 78; tongue and skin as before; pain in head, giddiness, tinnitus aurium; muscular debility is and has been very great; epistaxis; no pain in neck or chest; sense of fulness and tenderness of abdomen; one stool, fetid. ℞.—Pil. Hydrarg. pulv. Dover a. grs. ii, sexta. quad. hora.; mucilage for drink.

4th. 3 P. M.—No stool; pulse 75; respiration natural; skin and tongue as yesterday; neck and chest easy; abdomen scarcely distended but somewhat tender; pain in the top of the head; no tinnitus aurium; epistaxis; feet disposed to be cool. ℞.—Infus. senna; warm fomentations to head; mucilage for drink; keep the feet warm.

5th—Much as yesterday except head easy.

6th. 2 P. M.—One stool consistent, mixed with mucous, fetid; some pain in the head; no tinnitus aurium; considerable uneasiness in the back; pain in the bowels; pulse 80; respiration 20; feet more disposed to coldness ℞.—Pil. Hydrarg. grs. iiss; tart. ant. grs. 1-10, to be taken immediately, and repeated at intervals of four hours; a large poultice to abdomen; warm applications to forehead. At 4 o'clock the pill had vomited and moved the bowels, discharge thin, cider colored, fetid; pain in head and abdomen abated. Discontinue the pills; and rub the back with laudanum and ammonia.

7th. 12 M.—Easy and comfortable; one stool, thin, fetid; pulse 68; respiration 17, regular; tongue less red. ℞.—Mucilage and gruel.

8th. 12 M.—Easy and comfortable; one stool; pulse 76, (perhaps occasioned by her having just taken a little nourishment;) respiration and countenance indicative of comfort; some appetite.

In this case much benefit seemed to follow the vomiting occasioned by the pill of blue-mass and tartar on the 6th. This is the case in which memory was lost during my whole attendance.

*Case* 3. James Tucker, aged 25, here three weeks, called at my office on the 5th of June, 1847, complaining of great muscular weakness; countenance pallid; pain in the head and back; bowels regular; no appetite; tongue slightly furred white. ℞.—Emetic of ipecac., which vomited him well, but did not act on the bowels.

8th.—Countenance more expressive of indisposition, still very weak; bowels costive; otherwise much as before. ℞.—Calomel. Pil. Hydrarg. aa. grs. x, opii grs. i.

9th.—Pulse 90; skin soft; little tenderness of abdomen; respiration natural; tongue, countenance as yesterday; medicine has not operated. ℞.—Sulph. magnes. ℥ii.

10th.—Pulse, morning, 98, evening 104; skin perspiring, warm; tongue smooth, red, and small; tenderness at epigastrium and right iliac-fossa, indeed, of right side of abdomen generally; six or eight stools, fetid, watery; feeling "right smart." ℞.—Suppository of opium after each liquid stool, until they are reduced to one in six hours. Arrow-root for diet, mucilage for drink. ℞.—At night pil. Hydrarg. grs. xv, ip. opii aa. gr i.

11th.—But one stool; skin and respiration natural; pulse 84; tongue as yesterday; no nausea; pain in abdomen; slept well.

12th.—Pulse 96; skin and respiration natural; head slightly uneasy; tongue unchanged; throat sore and red; one stool; abdomen tender and distended. ℞.—Seidlitz powder to be taken at 11 A. M.; gargle of capsicum, eve. Medicine operated five or six times, thin, yellow, fetid; pulse 90, skin natural temperature. ℞.—Suppository of opium after each watery stool.

13th—Tongue red, aphthous; throat thought to be less sore; two stools, yellow, fetid; abdomen not distended, but slightly tender; pulse 96; skin natural; slight numbness of head. ℞.—Pil. Hydrarg. grs. viii, pulv. Dover grs. v.

14th.—Much as yesterday, no medicine.

15th.—Five or six discharges from bowels, watery, fetid, yellow; tongue broader, still red; no tympanites; some tenderness of abdomen; skin less warm; pulse 100. ℞.—Suppository of opium.

16th.—No stool since the suppository; pulse 95; tongue less red; abdomen easy.

18th.—Decided improvement; pulse 87; tongue becoming more natural; no stool; no tenderness of abdomen, some appetite. ℞.—Injection of warm water; no medicine. Continued to improve.

*Case* 4. Sylla, aged forty-five, has been complaining for some time of capricious appetite and general indisposition. July 22, 1841, took an emetic of ipecac, which discharged some phlegm, but little bile.

26th.—Some chilliness; slight pain in the back, neck, and head; tongue whitish, broad, considerable muscular debility; skin moderately warm; pulse frequent; not full. ℞.—Calomel ℈i.

27th.—Medicine has operated several times; stools thin and watery, slightly bilious. But little if any change in general appearance. ℞.—Calomel ℈ii.

28th.—Medicine has operated very much as before; skin not hot but dry; giddiness of head; sore throat upon swallowing; pulse 110 small; tongue disposed to be clammy. ℞.—Calomel ʒi.

29th.—Medicine operates frequently, stools small, watery, fetid; muscular debility great; skin dry, not hot; tongue as yesterday; does not complain of nausea; giddiness, and tinnitus aurium. No medicine.

30th.—Much as yesterday, except that the pulse is becoming quicker and weaker; bowels frequently moved, stools of same color; feet cool.

31st.—Tongue very white; skin dry and warm; little uneasiness upon pressing the abdomen; great muscular debility; still some pain in the back of the neck and singing in the ears; pulse 120; weak; diarrhea continues. ℞.—Emp. episp. to abdomen. Pil. Hydrarg. grs. v, thrice a day; enema of starch and laudanum, gtt. 20, after each liquid stool. Mucilage for drink, arrow root for diet.

August 2.—Tongue is casting its fur; red and clammy, skin very much as before; stools somewhat less frequent, but of the same character; some tenderness upon pressing the abdomen, but no tympanites perceptible. ℞.—Pil. Hydrarg. grs. v, night and morning.

4th.—Tongue has cast off a good deal of the fur, but is less red, and very much disposed to be dry; roaring in the ears, and soreness of throat unabated; bowels less disposed to diarrhea; had two stools, which from description, seem to have been consistent bilious ones. Is considerably stupid, which is attributed to the use of laudanum last night. She had a serous stool for which injection of fifteen drops laudanum was given and immediately returned. Thirty were given and retained. No meteorism. Somewhat deaf. ℞.—Pil. Hydrarg., night and morning.

5th.—Very much as yesterday, except that she has had no stool. ℞.—Injection of warm water to move the bowels, which brought two watery yellowish motions. ℞.—Pil. Hydrarg. grs. v, at night.

7th.—Pulse 112; countenance brighter, but delirious at night; tinnitus aurium continues; and soreness of throat when she swallows; bowels continue loose, six or eight stools daily, watery, yellow, and fetid. Skin nearly of natural temperature on the body, but feet cool; tongue dry. ℞.—Continue the medicine. Add a spoonful of wine to each cup of arrow-root. Pediluvia and warm brick or sinapisms to feet as may be necessary.

10th.—Seems to be slightly improving; countenance better; pulse 110, but weak; skin of natural temperature on body, but dry, cool feet; tongue still dry; bowels still loose, will not retain the injection of starch and laudanum. Talks less incoherently. ℞.—Suppository of opium in lieu of the injection. Continue the arrow-root with wine; mucilage for drink. A blue pill every other-night.

15th.—Remains very much the same, except that the bowels are less disposed to diarrhea—indeed it was deemed advisable to-day to give an enema to move the bowels, which caused excruciating pain. Some tenderness of abdomen; for the last two days gurgling can be produced by agitating the right iliac region; pulse ranging from 112 to 120 small. Skin not particularly dry, and of good temperature, but feet cool; does not complain of head; tongue still dry, and covered with a granular coat, like half dried mortar; throat said to be less sore. Continue medicines and diet. If it should be necessary to move the bowels, a small quantity of ol. Ricini. Blisters have been kept discharging on the abdomen.

19th. She remained very much as last reported the next day, except that she seemed to be touched with mercury, breath slightly tainted, and the parotid gland began to swell; in consequence of which the blue pill was discontinued. It has been necessary to give one small dose of oil (f℥ii) to move the bowels. By some strange mistake, or else to try a project of his own, her husband introduced a candle up the rectum. When it was taken out, it was found smeared with blood. There was some difficulty of swallowing, though she complained of no soreness of throat. Of late, has had little disposition to talk, but appears rational. Her strength was thought to have improved, and yesterday morning she sat up on the bedside and washed her face and hands; but when I arrived, at 11 o'clock, she seemed to be dying. Her extremities were cold, and her body covered with a clammy perspiration; respiration short, and pulse scarcely perceptible. Friction to the jaws had appeared to diminish the swelling of the parotid glands. By stimulants internally and sinapisms externally, on yesterday, she rallied considerably, but sank and died to-day at 12 o'clock.

This was among the first lot of cases which I recognised as typhoid fever. In the commencement of the case, I acted upon my experience in bilious fevers, and increased the dose of calomel to procure consistent bilious stools; seeing, at the same time, that the form of fever was materially different from ordinary bilious fever. Whether the administration of these large doses of calomel exerted any decided influence of injurious tendency, I cannot with any assurance say. One thing is true: the pulse became rapid much quicker than it usually does. Though I have seen it undergo that change still more rapidly, when it could not be attributed to calomel, or any other mode or treatment. It satisfied me that large doses of calomel were not *the* remedy in this kind of a case. This case, exhibits, too, the want of correspondence between mercurializing and improvement, as the patient continued to grow worse during its inception and progress. Appearances on dissection are given already.

*Case* 5. Joel Holden, aged 27, of low stature, but very corpulent, has been complaining four days of general indisposition; some general chilliness and feverishness; had taken three doses of extract of white walnut, which had procured "consistent dark bilious stools," as represented by him; also, an emetic of ipecac. At present (May 9th, 1847), pain in head and back, face flushed; pulse 88; tongue furred, whitish, broad, indented by teeth; some diarrhea; no tympanites or tenderness of abdomen; considerable restlessness and anxiety; temperature of body increased; skin not harsh; respiration natural, muscular weakness. ℞. Calomel, gr. xii., pulv. Dover, gr. x. M.

10th. Very mnch as yesterday; very restless, anxious, and of evil foreboding; skin warm, but not harsh. ℞. Calomel, pil; hydrarg. a gr. v.; ipecac, gr. i; op. gr. $\frac{1}{4}$.

11th. Pulse 90, rather full; respiration natural, except occasional sighing; tongue rather more white, broad; constant screatus; very uncomfortable sensations referred to the epigastrium; four stools, thin and watery; no tympanites or marked tenderness; heat of surface somewhat increased, skin dry; pain in the back. ℞. spts. nitre ʒi, ipecac, gr. i, every two hours. Back to be rubbed with laudanum and ammonia. If bowels become troublesome, enema of starch, with 20 drops of laudanum after each stool, until reduced to one every six hours.

12th. Less pain in back; four stools, bright yellow, of rather more consistence—of thin molasses. The ipecac vomited him; threw up phlegm, mixed with a little bile; skin still warm, face flushed; rather less distress at epigastrium, but still restless; tongue continues whitish, something more red at the tip and sides; no tenderness or meteorism. ℞.—Calomel, pil hydrarg., a gr. x, morphia gr. ¼.

13th. Pulse 94, skin warm over whole surface; face flushed, sometimes one cheek, sometimes both; tongue very much the same, screatus continues; complains of some soreness of throat; upon examination, it was found somewhat red; still much uneasiness of epigastrium; three stools, same character; slight tympanites, urine of good color and ordinary quantity. ℞.—Ipecac, gr. i, every hour during the febrile excitement during the evening. Mucilage with carb. soda for drink; a blister to epigastrium to-night, sponging body with cold water during the fever; early to-morrow morning, pil. hydrarg., gr. x, quinine gr. iv, sulph. morph., gr. ⅛; gargle of capsicum.

14th. Pulse 96, skin warm; perspired freely yesterday; tongue very much as before, perhaps a shade more red; sore throat, screatus continues; less oppression at epigastrium, in consequence of which the blister was not applied as directed; three stools, still deep yellow, and same consistence; immense discharge of flatus during defecation; no tenderness of abdomen, but still slight meteorism; some pain in head, and thought to have been somewhat delirious last night; respiration somewhat irregular; sighing. ℞.—Repeat the blue mass and quinine to-morrow morning; blister to back of neck to-night. Continue the sponging whilst the heat of body is considerable.

15th. Blister drew well, but the head is very little relieved; two stools, same character; considerable discharge of flatus; black powder in bottom of vessel; tongue much the same; throat more sore, somewhat ulcerated; screatus continues; some nausea last night; skin still warm; lips remain plump, abdomen as yesterday; pulse 96. ℞.—Gargle of nitrate potash, brandy and honey. Continue drinks and sponging; no medicine. Cut off the hair.

17th. Delirium is increasing, notwithstanding the blister discharges freely; face continues flushed, lips plump, tongue more red, throat no better; respiration irregular, abdomen as last stated; two stools, much flatus; countenance becoming more anxious; pulse 100. ℞.—Pill hydrarg., pulv. Dover a gr. iii, sexta quaque hora. Carb. ammonia, gr. ii, secunda quaq. hora in mucillage.

19th. Pulse 105; [it is generally, of late, from 10 to 15 more frequent at night than at my visit,] skin still above the natural temperature. On the thorax is an eruption, which seems an effort at the rose-colored spots; at least there are some fifteen or twenty tawny spots, of about the size, slightly elevated. Tongue broad, brown, red at tip and edges; complains less of throat, though it is still ulcerated; screatus continues; tympanites, but no tenderness; two stools, unchanged, flatus excessive; urine of good color; more delirious, still restless; on 18th perspired freely. Of late emaciation has progressed con siderably; and the countenance is becoming sharpened. ℞.—Continue the medicines, emp. episp. to epigastrium.

21st. In every respect worse; countenance more sharpened; but the lips have retained their plumpness throughout his illness; tongue more brown, but moist and broad, red at edges and tip; two stools daily; skin warm; no change in the spots; delirious night and day; pulse 130.

23rd. Becoming comatose; eyes suffused; tongue still moist but dark, meteorism; has had no stool for sixty hours; pulse too quick and tremulous to be counted with certainty. I left him with the belief that he would not live more than twelve or eighteen hours; but he did live between two and three days, sinking very gradually, without having another motion from his bowels.

In this case, medicines did not appear to exert any influence whatever. The fecal discharges were all the time of a rather bright yellow color, differing little in color or consistence from healthy bile; and the diarrhea was at no time very troublesome. The febrifuges appeared to mitigate the fever at the time of administration, but next day it was at least as high as ever. Sponging was very grateful, but otherwise seemed of no use. Quinine had no effect in postponing or mitigating the afternoon exacerbation. Perhaps if it had been doubled or trebled it might have done better. Blisters drew well, and discharged well, but seemed to produce no effect. Restlessness was very great, yet almost every night he would get some comfortable sleep, either in the early or latter part of the nignt.

